# Renal Interstitial Invasion by Group A *Streptococcus*: A Rare Presentation

**DOI:** 10.1155/2022/5881375

**Published:** 2022-05-09

**Authors:** Francis Lomanta, Shankar Upadhyayula

**Affiliations:** ^1^Department of Pediatrics, Akron Childrens Hospital, Akron, OH, USA; ^2^Division of Infectious Diseases, Akron Childrens Hospital, Akron, OH, USA

## Abstract

We report the case of a 5-year-old who had interstitial invasion of his kidneys with group A *Streptococcus* (GAS). Glomeruli and tubules were relatively preserved. He recovered from this event and was admitted a couple of months later with dilated cardiomyopathy needing a heart transplant. To our knowledge, this is the first reported case of direct invasion of renal interstitium by GAS.

## 1. Background

*Streptococcus pyogenes* also known as GAS is a Gram-positive extracellular bacterium that is a common human pathogen. It usually causes mild infections such as pharyngitis, impetigo, and scarlet fever. Invasive group A streptococcal infections (defined as those in which GAS infects a normally sterile site) are uncommon, although serious with a high case fatality rate [1]. Incidence of invasive GAS (iGAS) infection is typically higher in winter and spring and lowest in autumn. Risk factors for iGAS seem to vary by age. In adults (18 to 44 years of age), exposure to one or more children with a sore throat, HIV, and a history of injection drug use seem to be important associations. In older adults who are >45 years of age, number of persons in the home, diabetes, cardiac disease, cancer, and corticosteroid use seem to be most relevant [2–5].

Reports suggest a notable global increase in the incidence of iGAS disease since the late 1980s [6]. World Health Organization (WHO) ranks GAS as the ninth leading infectious cause of human mortality, with a significant portion of deaths being attributable to invasive infections. The overall case-fatality rate of invasive GAS is estimated to be from 10% to 15% with much higher rates for streptococcal toxic shock syndrome [5]. GAS strains are defined by the presence of the group A carbohydrate on their surface and are further classified based on serological or genetic differences in the surface M protein, encoded by the *emm* gene. Over 220 different *emm* types have been documented and remarkable differences in *emm* type prevalence exist in different populations. In particular, the dominant circulating *emm* types in industrialized populations are usually different and much less diverse than those that circulate in socioeconomically disadvantaged populations [5].

The M protein seems to be a major virulence factor for the organism. In clinical reports and epidemiological studies of invasive and toxic streptococcal diseases, M types 1, 3, 11, 12, and 28 have frequently been reported, with M1 and M3 being the most common [7,8]. The M protein resists phagocytosis by neutrophils by interfering with the complement pathway in many different ways. Secreted virulence factors called exotoxins are also important in the pathogenesis of GAS-related illnesses. These include extracellular pyrogenic exotoxins A, B, and C that cause hypersensitivity including rash and acute interstitial nephritis. Additional secreted factors include exotoxin F (mitogenic factor) and streptococcal superantigen (SSA) 1 that stimulate T cells by directly binding class II molecules on antigen-presenting cells (dendritic cells, B cells, and macrophages) to the T-cell receptor. This less specific interaction leads to the stimulation of a large proportion (up to 20%) of all circulating T cells and a consequent release of proinflammatory cytokines from T cells and other cells of the immune system [4,8]. This intense inflammatory cascade is responsible for the clinical features of streptococcal toxic shock syndrome [8,9]. It seems evident that the virulence factors act coordinately during the various stages of infection, colonization, invasion, spread, and pathogenesis [10]. The explanation for why invasive disease occurs in some people and not others when they are exposed to GAS is not clear. Circulating types may acquire virulence factors as a result of horizontal gene transfer events that permit them to invade sterile sites, but host and environment factors likely play an important role in susceptibility and remain a research priority [6].

The incidence of invasive disease in developed countries is currently estimated at about 3–4/100,000 (∼10,000 cases per year) [7]. Interstitial invasion of the kidney by GAS has not been reported previously. Whether this unusual presentation is a host characteristic or due to a certain type of GAS is unclear.

## 2. Case

A 5-year-old African-American male with no significant past medical history presented to the emergency department (ED) with fever, myalgias, diffuse rash, and abdominal pain. He started feeling unwell three days prior with fever, decreased activity, and poor appetite. One day prior, he had an episode of nonbloody diarrhea as well as nonbloody and nonbilious emesis. On the day of presentation, he developed a diffuse maculopapular rash (sandpaper-like) after which he was brought to the ED. There were no reported sick contacts, no known COVID-19 exposures and no cough, congestion, or shortness of breath.

In the ED, he was noted to be ill-appearing, lethargic, febrile (101.8°F), and tachycardic (heart rate: 150–160 beats/minute) with normal blood pressure. He was also noted to be tachypneic (respiratory rate >30 per minute). Laboratory evaluation (additional data in Table 1) was notable for the following: respiratory viral panel (Biofire® FilmArray) positive for rhinovirus/enterovirus, negative SARS-CoV-2. Sodium 117 (normal 133–145 mEq/L), chloride 83 (normal 96–108 mEq/L), blood urea nitrogen 78 (normal 4–19 mg/dL), creatinine 3.14 (normal 0.3–0.5 mg/dL), albumin 2.7 (normal 3.2–4.5 g/dL), lactate 2.9 (normal 0.5–1.6 mmol), white blood count 34.8 (normal 5.5–15.5 10E9/L), absolute neutrophil count 32.4 (normal 1–7 10E3/*μ*L), and C-reactive protein (CRP) 42.1 (normal 0.0–1.0 mg/dL). Urinalysis showed 3+ hemoglobin and 3+ protein. Chest radiograph, abdominal radiograph, and an ultrasound scan of the abdomen were performed and reported to be unremarkable. He received fluid resuscitation with 40 cc/kg of normal saline and was started on ceftriaxone and vancomycin pending culture results.

**Figure 1:**
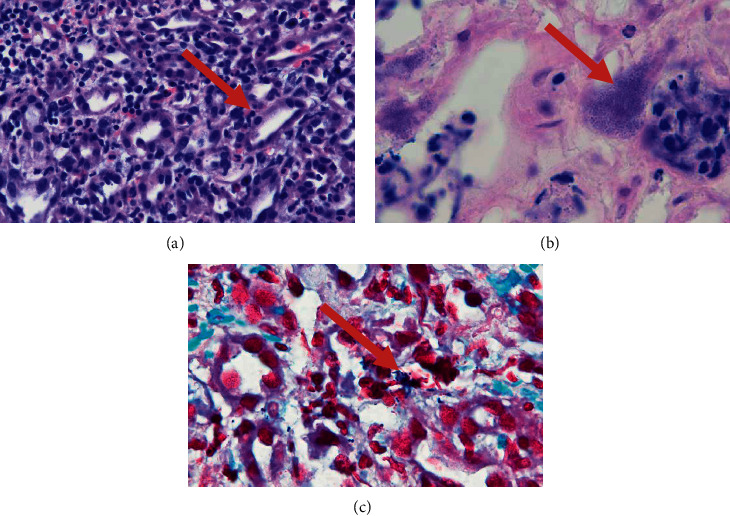
(a) Heavy neutrophil infiltrate in the interstitium with the sparing of the tubular lumen. (b) Clusters of gram-positive cocci in the interstitium. (c) H&E stain showing gram positive cocci in pairs and chains.

After this, he was admitted to the pediatric intensive care unit (PICU). Additional labs in the PICU showed a procalcitonin of >100 (normal <0.10 ng/ml), ferritin of 946 (normal 12–113 ng/ml), sIL2-R (soluble interleukin 2 receptor) levels of 20417 (normal ≤2,126 U/mL), triglyceride of 596 (normal 0–74 mg/dl), and fibrinogen of 509.5 (normal 150–410 mg/dl). The patient remained normotensive and on room air throughout his PICU course. Blood and urine cultures returned positive for GAS. At this time, patient's presentation was felt to be consistent with streptococcal toxic shock syndrome. Hemograms were trended and showed profound anemia and thrombocytopenia. He had progressive decline of his renal function with decreasing urine output and worsening creatinine to 4.16 mg/dl (normal 0.3–0.5 mg/dL). Due to worsening renal function, he was placed on renal replacement therapy and a renal biopsy was obtained to rule out a rapidly progressing glomerulonephritis. Biopsy findings revealed marked acute and focal suppurative inflammation of renal interstitium with intracellular Gram-positive cocci in chains and abundant extracellular cocci in chains and clusters in addition to numerous bacterial colonies surrounded by suppurative acute inflammation. Edematous interstitium as well as ectatic peritubular capillaries was seen throughout the specimen. No pyelonephritis/tubulitis was identified. Although focal tubules demonstrated necrosis, no definitive gangrenous necrosis of tubules was seen. No abscesses or epithelial crescents were identified. The inflammatory infiltrate widely separated glomeruli, vasculature, and tubules but did not significantly involve renal vasculature, tubules, or glomeruli. No electron-dense deposits were identified within glomeruli which demonstrated normal foot processes and normal glomerular basement membranes. The findings were consistent with the invasion of the interstitium by *Streptococcus* species (Figure 1). The patient stayed in PICU for 5 days, before he was transferred to the floor.

The subsequent in-hospital stay was complicated by recurrent bouts of unexplained fevers, left elbow joint effusion, and anemia. Infectious evaluation was repeatedly negative. Magnetic resonance imaging (MRI) of his abdomen for fever evaluation showed renal papillary necrosis, but no abscess. Postinfectious autoimmune or autoinflammatory processes were felt to be likely. Rheumatology was consulted. Repeat inflammatory markers were elevated. sIL2-R levels were repeated and down trending at 2489 U/ml. Rheumatology felt that patient's constellation of symptoms plus ongoing inflammation was most consistent with systemic-onset juvenile idiopathic arthritis in the setting of his recent GAS infection. The patient was started on a course of prednisone, and his clinical course improved with the steroids. He was discharged home after 54 days in the hospital with a prednisone taper and antihypertensive therapy with amlodipine. One week following discharge, the patient followed up with rheumatology and nephrology. Other than hypertension, he was well, and this was thought to be due to noncompliance with antihypertensive therapy. Follow-up labs were stable and reassuring (creatinine level was 0.73 mg/dl, CRP was 1.1 mg/dl, and ESR was 50 mm/hr). He was prescribed a 1-month prednisone taper and amlodipine refill. His next documented follow-up was 2 months later by when he completed his prednisone taper. He was no longer taking amlodipine due to his prescription running out, but by now, he was normotensive and so his amlodipine was discontinued. The creatinine level at that time was 1.10 mg/dL.

One month after his last clinic visit, i.e., 4 months from his initial presentation, he developed upper respiratory symptoms with cough and increased work of breathing and returned to the ED. He was afebrile but tachycardic and tachypneic. The respiratory viral panel was once again positive for rhinovirus/enterovirus. Chest radiographs showed diffuse ground glass opacities, bilateral pleural effusions, and mildly prominent cardiac silhouette. EKG showed right and left ventricular hypertrophy. The lab workup showed troponin-T 38 ng/L (normal 0–14 ng/L) and brain natriuretic peptide (BNP) 81,775 (normal 0–125 pg/mL). Echocardiogram showed a shortening fraction of 9% (normal 28–46%), ejection fraction of 19% (normal 56–78%) with moderate mitral and tricuspid regurgitation, and increased end-diastolic dimension. His presentation was felt to be most consistent with myocarditis/cardiomyopathy. He was admitted to the PICU and required inotropic support with milrinone to optimize perfusion. Respiratory status was stable to improving. Inflammatory markers were unremarkable with CRP 1.7 mg/dl, procalcitonin 0.14 ng/dl, ferritin 895 ng/mL, and sIL-R 697 U/ml. Infectious workup for myocarditis was negative. Because this patient had two unusual and severe episodes of illness immunology was consulted who felt that direct genetic testing for autoinflammatory disorders would be the favored approach. His cardiac status did not improve despite support in the ICU and after further discussion with our cardiology team, he was transferred to a transplant center. The transplant center gave him a diagnosis of idiopathic dilated cardiomyopathy and he received an orthotopic heart transplant soon after.

## 3. Discussion

Our patient was a previously healthy 5-year-old male who was admitted to the PICU with systemic inflammatory response syndrome and rapidly progressing acute kidney injury requiring renal replacement therapy. The severity of the patient's initial symptoms warranted a broad workup. Differential diagnoses included severe sepsis, MIS-C (multisystem inflammatory syndrome in children related to COVID-19), Kawasaki disease with shock, hemolytic uremic syndrome, hemophagocytic syndromes, etc. He was noted to have invasion of renal interstitium with GAS following a renal biopsy. Streptococcal infection-associated ARF is most commonly attributable to sepsis-related ischemic acute tubular necrosis or acute interstitial nephritis or acute poststreptococcal glomerulonephritis. However, direct interstitial invasion due to GAS is likely very rare.

Literature search was completed on PubMed for review articles and case reports (in the last 20 years) on invasive GAS infections with renal involvement. Several reviews of invasive GAS infections have been published and a few case reports of interstitial nephritis in patients with GAS bacteremia, but there were no reports of direct interstitial invasion due to GAS [1–5,10–12].

Chang et al., reported a case of a 22-year-old previously healthy male who developed a sore throat, myalgia, and diarrhea. His workup was significant for GAS bacteremia. He developed hypotension and acute kidney injury requiring four sessions of hemodialysis. He then underwent a kidney biopsy that showed acute interstitial nephritis with eosinophilic invasion. Immunohistochemical study depicted diffusely strong positive signals of anti-streptococcal pyrogenic exotoxin B (SPE B) antibodies in tubular epithelial cells and tubulointerstitial compartments of the cortical tissue [11].

Binkhorst et al., described a 3-year-old girl who presented to the ED with fever, vomiting, diarrhea, sore throat, bilateral purulent conjunctivitis, and a red tongue. A week prior to presentation, she had a low-grade fever, a sore throat, a red tongue, and bilateral purulent conjunctivitis. In between these two episodes, there was a relatively symptom-free interval. Work-up was significant for GAS bacteremia, proteinuria, hematuria, and elevated creatinine. Renal biopsy showed diffuse hemorrhagic interstitial nephritis. Her kidney function improved, and she did not require hemodialysis [12].

Our patient was bacteremic, similar to those reported by Chang et al. [11] and Binkhorst et al. [12]. There was also evidence of interstitial inflammatory infiltrate, but what was unique was the direct invasion of the renal interstitium by GAS.

Bjorck et al. in their observational study report that critically ill patients with invasive GAS (iGAS) infection had a lower mortality risk but a higher degree of renal failure compared to similarly ill sepsis patients. The authors note that patients with emm1/T1 demonstrated more circulatory and renal failure than patients with other serotypes of iGAS [3].

Limitations of this case report include our inability to complete serotyping on the GAS isolate and autoinflammatory syndrome evaluation to understand this unusual pattern of illnesses. Nevertheless, this presentation was unique.

At the time of writing this report, the patient is more than a year out from his initial presentation. He is stable and is following up with the cardiac transplant center. The unusual course of his illnesses from the time of onset of iGAS infection leading up to heart transplant raises questions about unique host characteristics. Genetic testing for autoinflammatory disorders has not yet been completed but anticipate will happen going forward.

## 4. Conclusion

GAS invasive infection of the renal interstitium has not been previously reported to our knowledge. In patients presenting with iGAS infection with severe and progressive acute kidney injury, interstitial invasion due to GAS should be considered.

## Figures and Tables

**Table 1:** Lab work at admission and upon recovery.

Lab tests with normal ranges in parenthesis	On admission	Upon recovery
C-reactive protein (0.0–1.0 mg/dl)	42.1	0.9
Procalcitonin (<0.1 ng/ml)	>100	0.22
Complete blood count	WBC 34.8	WBC 12.6
WBC (5–15 10E9/L)	HGB 11.0	HGB 8.9
HGB (11.5–13 g/dL)	HCT 29.1	HCT 25.9
HCT (34%–39%)	PLT 169	PLT 370
Platelet 250–550 10E9/L)		
Iron (45–160 *μ*g/dL)	<5	26
TIBC (228–428 *μ*g/dL)	139	206
% Saturation (9–55%)	4	13
Ferritin (12–133 ng/ml)	946	545
Serum IL-2R (</ = 2,126 U/mL)	20417	2489
Triglyceride (0–74 mg/dl)	596	147
Fibrinogen (150–410 mg/dl)	509.5	319.7
Blood and urine culture	Positive for group A *Streptococcus*	
Kidney biopsy	Gram-positive cocci in chains	
Epstein barr virus PCR	Negative	
ANA	Negative	
C3 (77–143 mg/dl)	61	
C4 (7–40 mg/dl)	4	
SARS-CoV-2 PCR	Negative	
